# Biomechanical characterization of TIM protein–mediated Ebola virus–host cell adhesion

**DOI:** 10.1038/s41598-018-36449-2

**Published:** 2019-01-22

**Authors:** Matthew A. Dragovich, Nicole Fortoul, Anand Jagota, Wei Zhang, Krista Schutt, Yan Xu, Michelle Sanabria, Dennis M. Moyer, Sven Moller-Tank, Wendy Maury, X. Frank Zhang

**Affiliations:** 1 Department of Mechanical Engineering & Mechanics, Lehigh University, 19 Memorial Drive West, Bethlehem, PA 18015 USA; 2 Department of Chemical and Biomolecular Engineering, Lehigh University, 111 Research Drive, Bethlehem, PA 18015 USA; 3 Department of Bioengineering, Lehigh University, 111 Research Drive, Bethlehem, PA 18015 USA; 4 Department of Microbiology, University of Iowa, 51 Newton Rd, Iowa City, IA 52242 USA; 5 Present Address: Department of Medicine, Rheumatology, Columbia University Medical Center, New York, NY 10032 USA; 6 Present Address: Preclinical Research, Cresilon, 122 18th Street, New York, NY 11215 USA; 7 Present Address: Acoustics division, Apple Inc., One Apple Park Way, Cupertino, CA 95014 USA

## Abstract

Since the most recent outbreak, the Ebola virus (EBOV) epidemic remains one of the world’s public health and safety concerns. EBOV is a negative-sense RNA virus that can infect humans and non-human primates, and causes hemorrhagic fever. It has been proposed that the T-cell immunoglobulin and mucin domain (TIM) family proteins act as cell surface receptors for EBOV, and that the interaction between TIM and phosphatidylserine (PS) on the surface of EBOV mediates the EBOV–host cell attachment. Despite these initial findings, the biophysical properties of the TIM-EBOV interaction, such as the mechanical strength of the TIM-PS bond that allows the virus-cell interaction to resist external mechanical perturbations, have not yet been characterized. This study utilizes single-molecule force spectroscopy to quantify the specific interaction forces between TIM-1 or TIM-4 and the following binding partners: PS, EBOV virus-like particle, and EBOV glycoprotein/vesicular stomatitis virus pseudovirion. Depending on the loading rates, the unbinding forces between TIM and ligands ranged from 40 to 100 pN, suggesting that TIM-EBOV interactions are mechanically comparable to previously reported adhesion molecule–ligand interactions. The TIM-4–PS interaction is more resistant to mechanical force than the TIM-1–PS interaction. We have developed a simple model for virus–host cell interaction that is driven by its adhesion to cell surface receptors and resisted by membrane bending (or tension). Our model identifies critical dimensionless parameters representing the ratio of deformation and adhesion energies, showing how single-molecule adhesion measurements relate quantitatively to the mechanics of virus adhesion to the cell.

## Introduction

Ebola virus (EBOV) disease is a severe and often fatal illness in humans. First identified in 1976, and with a fatality rate of 50 to 70%, the disease has caused about 15,000 deaths^1–4^. EBOV is a filamentous, enveloped, non-segmented, negative-sense RNA virus that belongs to the virus family Filoviridae. Filoviruses, such as EBOV, have an extensive tissue tropism. Dendritic cells and macrophages are considered to be their first targets. Subsequent rounds of infection follow in a variety of cell types including epithelial cells such as hepatocytes, stromal cells and to a lesser degree endothelial cells^5,6^. There are five closely related species: Ebola virus (EBOV, formerly Zaire ebolavirus), Sudan ebolavirus (SEBOV), Taï Forest ebolavirus (TAFV), Reston ebolavirus (REBOV), and the proposed most recent addition, Bundibugyo ebolavirus (BDBV)^7^.

The EBOV genome encodes seven structural proteins, nucleoprotein (NP), polymerase cofactor (VP35), matrix protein (VP40), glycoprotein (GP), replication-transcription protein (VP30), minor matrix protein (VP24), RNA-dependent RNA polymerase (L) and two secreted non-structural glycoprotein (sGP and ssGP)^5^. In the first step of EBOV lifecycle, viral attachment through interaction between cellular molecules is followed by endocytosis, including macropinocytosis^8^. Subsequent trafficking of the virion through the endosomal compartment to the late endosomal/lysosomal compartment results in viral-endosomal membrane fusion and release of the viral ribonucleoprotein complex into the cytoplasm. Transcription of the negative-sense viral RNA genome by the viral polymerase complex yields mRNAs that are translated by cellular ribosomes. Upon replication, viral RNAs and structural proteins such as VP40 and GP are assembled at the plasma membrane into enveloped virus particles that bud from the host cell’s surface^5,9,10^, thus repeating the cycle and spreading the virus.

T-cell immunoglobulin mucin domain 1 (TIM-1) is a type 1 transmembrane glycoprotein and a member of the TIM family^11^. The TIM proteins are phosphatidylserine (PS) receptors, binding to PS on the surface of apoptotic bodies and clearing these dead cells from circulation. TIM-1 has also been recently recognized to enhance entry of an expansive range of viruses, including members of the picornavirus, filovirus (such as EBOV)^12^, flavivirus, alphavirus, arenavirus, and baculovirus families^13–15^. In addition to TIM-1, TIM-4, another TIM family member, has been shown to augment EBOV entry comparably to TIM-1^16^.

For enveloped viruses, where the capsid is surrounded by a lipid bilayer that contains the viral proteins, this enhancement is believed to occur through TIM binding to PS on the viral envelope. By hijacking the cellular mechanisms utilized in the uptake of apoptotic bodies mediated by TIM, EBOV is internalized into the host cell’s endosomes. EBOV internalization by TIM-1 is found to be solely PS-dependent, and does not require the presence of the viral surface glycoprotein^11,17^. This mechanism, known as apoptotic mimicry was first described for the vaccinia virus^18^. Consequently, TIM-1, TIM-4 as well as other PS receptor complexes such as Gas6/Axl, were identified as cellular proteins engaged in this process^19,20^. This class of viral receptors is known as PS-mediated virus entry-enhancing receptors (PVEERs)^20^.

Although TIM-1 and TIM-4 have been characterized as the PVEER for EBOV, little is known about the biomechanical properties of the TIM-1/-4 – host cell interaction that help to initiate EBOV internalization. In particular, the mechanical strength of individual interactions between TIM-1/-4 and EBOV, and how the mechanical interaction collectively drives virus adhesion, remain unclear. In this work, using atomic force microscopy (AFM)-based single-molecule force spectroscopy, a method where a single bond rupture between two molecules can be measured directly, we have quantified the mechanical strengths between TIM-1 or TIM-4 with the following interacting binding partners: PS, EBOV virus-like particle (VLP) and EBOV GP/vesicular stomatitis virus pseudovirion. As AFM can measure forces in the pN range, it is possible to investigate inter-molecular forces. This allows for even the weak interactions between tip-bound ligands and surface-bound receptor molecules to be quantified in terms of their affinities and rate constants^21^. Furthermore, AFM has been previously adopted to study virus-surface and virus-host cell interactions^22–24^. In the present study, we have demonstrated that the TIM-4–PS complex is more resistant to mechanical force than TIM-1–PS.

In order to relate the single-molecule cell receptor–virus particle binding to the behavior of the entire virus with the cell membrane, it is crucial to understand the mechanics of viral particle adhesion and engulfment. A number of approaches have been followed, including continuum, coarse-grained models, and even all atoms models^25–28^. Larger scale models such as triangulated membrane models and other continuum models can be used to study overall membrane adhesion without the high computational cost^29,30^. In this work, we wish to utilize on and off rates from single-molecule experiments to develop a continuum model of the virus-membrane adhesion process. Previously Chou *et al*.^31^ developed a model that is on the kinetics of virus binding and fusion, in particular on the competition between fusion and endocytosis. This discrete model was written in terms of on and off rates for individual processes that can be related to the adhesion energies that we will use in our model. However, the model does not explicitly include the effects of membrane stiffness and tension, which we show here to be critically important. Here, we have developed a simple biomechanical model to describe how the adhesive interaction between TIM and the viral surface drives EBOV adhesion to the host cell via a process involving membrane bending (and/or tension). A similar framework has been employed previously to study adhesion between nanoparticles and membranes^32–34^, and between vesicles and the plasma membrane^35^.

## Results

In this study, we characterized the mechanical strength that enables the EBOV-host cell pair to resist the forces of microenvironment. Because EBOV is classified as a BSL-4 pathogen, we elected to use an appropriate model system: virus-like particles (VLPs), which are non-pathogenic reagents that can be studied in BSL-2 labs. VLPs are prepared by over-expression of EBOV VP40 protein in mammalian cells. Expression of solely VP40 is sufficient to assemble and form VLPs that closely resemble the size and shape of EBOV^36^. Co-expression of VP40 with EBOV glycoprotein (GP) enhances the production of VLPs with glycoprotein present on their surfaces, making it an ideal model system to study EBOV entry^37^. The VLPs used in this study consisted of green fluorescent protein (GFP)-tagged VP40 and GP, derived from EBOV^15^. These VLPs have been shown to interact with and be internalized by host cells in a similar fashion as the native EBOV^12,15^. In addition to the EBOV VLP, we used an established pseudovirus model for EBOV. This pseudovirion was derived from a recombinant vesicular stomatitis virus encoding EBOV GP (EBOV GP/rVSV), and has been shown to accurately recapitulate the entry steps of wildtype EBOV^12,38^. A similar EBOV GP/VSV is currently used as a Ebola vaccine and undergoing phase 1 trials in Africa and Europe^39^.

**Figure 1:**
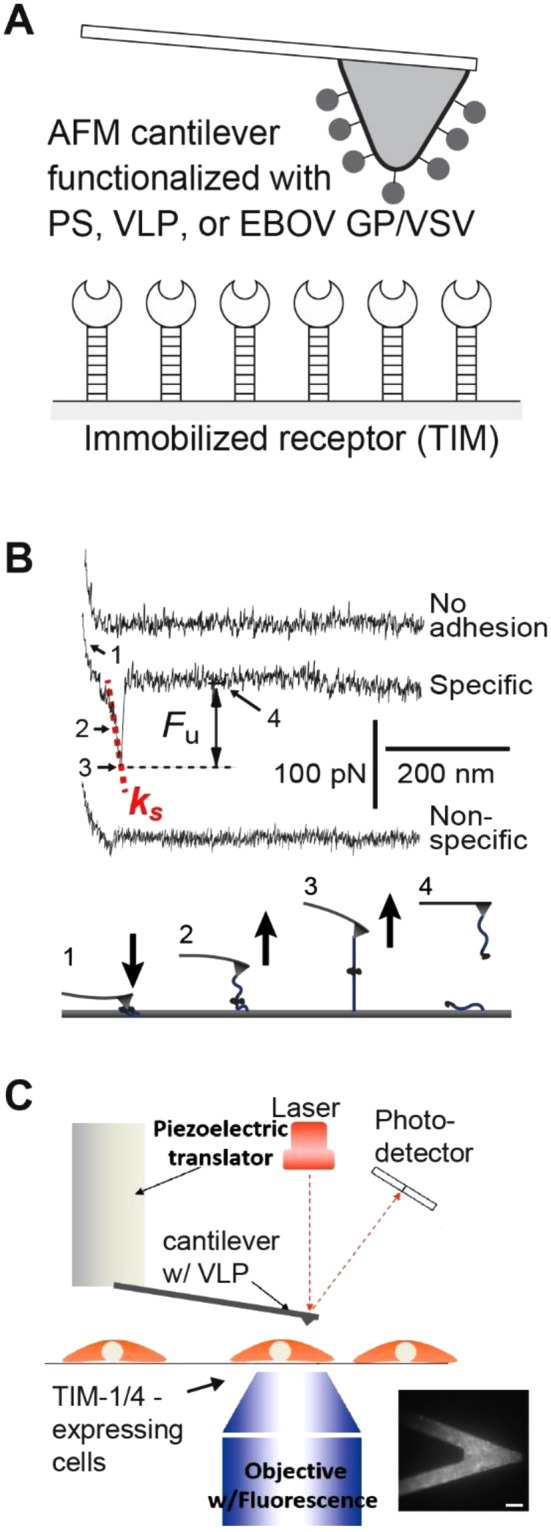
Schematic of the experimental system. (**A**) The AFM cantilever is functionalized with the PS, VLP, or EBOV GP/VSV. TIM-1 or TIM-4 is immobilized on the opposing glass surface. (**B**) The upper panel shows three sample AFM pulling traces of the TIM-4–PS interaction. The first (upper) trace involves no interaction. The second (middle) trace shows the rupture force of the TIM-4–PS complex. The lower trace shows a typical non-specific interaction. *F*_*u*_ is the unbinding force. *k*_*s*_ is the system spring constant and was derived from the slope of the force-displacement trace. The cantilever retraction rate of the measurements was 1.5 μm/s. The lower panel illustrates the four stages of stretching and rupturing a single ligand-receptor complex using AFM. (**C**) A schematic diagram of the live-cell AFM assay. Insert: micrograph of a VLP -functionalized cantilever showing GFP fluorescence. Bar is 20 µm.

For the AFM studies, PS-Polyethylene Glycol (PEG), VLP, or EBOV GP/VSV was covalently attached to the AFM cantilever. Immobilization of VLP was confirmed by fluorescence microscopic images of GFP on the AFM cantilever (Fig. 1C insert). Soluble recombinant TIM-1 or TIM-4 was covalently attached to a silanized cover glass (Fig. 1A).

All single-molecule force measurements were conducted using a custom-built AFM designed for operation in the force spectroscopy mode^40–42^. Using a piezoelectric translator, the functionalized cantilever was lowered onto a TIM-functionalized surface to allow binding between the TIM and PS (or other ligands) to occur. After a brief contact, the cantilever was retracted from the surface. Any binding interaction between TIM and ligand would lead to an adhesive pull-off force determined from the deflection of the cantilever via a position-sensitive two-segment photodiode. (Fig. 1A,B, lower panel).

Figure 1B shows three typical pulling traces. The first (upper) trace represents a majority (65–70%) of all the pulling curves, showing no interaction (i.e., no adhesive force) between the AFM tip and sample surface. The second (middle) trace, representing approximately 30% of the pulling curves in our single-molecule assay, shows the unbinding (i.e., pull off) force of the TIM-ligand complex (TIM-4–PS in this specific case). The unbinding force (*F*_*u*_) of the TIM-ligand complex is derived from the force jump that accompanies the unbinding of the complex. *k*_*s*_ is the system spring constant derived from the slope of the each pulling trace. The third (lower) trace, representing 5% (or less) of all pulling curves, shows a weak pull off force between AFM tip and surface. This typically occurred when one of the binding partners (TIM or ligand) is absent. We attributed these weak interactions to nonspecific interactions between the AFM tip and surface (Supplementary Figs S1 and S2). These nonspecific adhesion forces were significantly smaller than the unbinding forces of TIM-ligand interactions, averaged at approximately 18 pN and seldom exceeded 25 pN (i.e., mean + 1 SD). In addition, when the loading rate increases, the magnitudes of nonspecific interaction did not change significantly, whereas specific TIM-ligand unbinding forces increase with loading rates (Supplementary Figs S1 and S2).

To enable measurement of a single molecular interaction, the contact between the cantilever tip and the substrate was minimized by reducing both the contact duration (as low as 50 ms) and the compression force (~100 pN). The brief contact duration was chosen to ensure that, for the majority of contacts (67% or greater), no adhesion (rupture force) was observed between AFM tip and surface. Assuming the adhesion bond formation obeyed Poisson statistics, an adhesion frequency of ~33% in the force measurements implies that among the observed unbinding events, the probabilities of forming a single, double, and triple adhesion bonds between AFM tip and surface were 81%, 16%, and 2%, respectively^43^. Therefore, our experimental condition ensured there was a >80% probability that the adhesion event was mediated by a single bond^44^.

**Figure 2:**
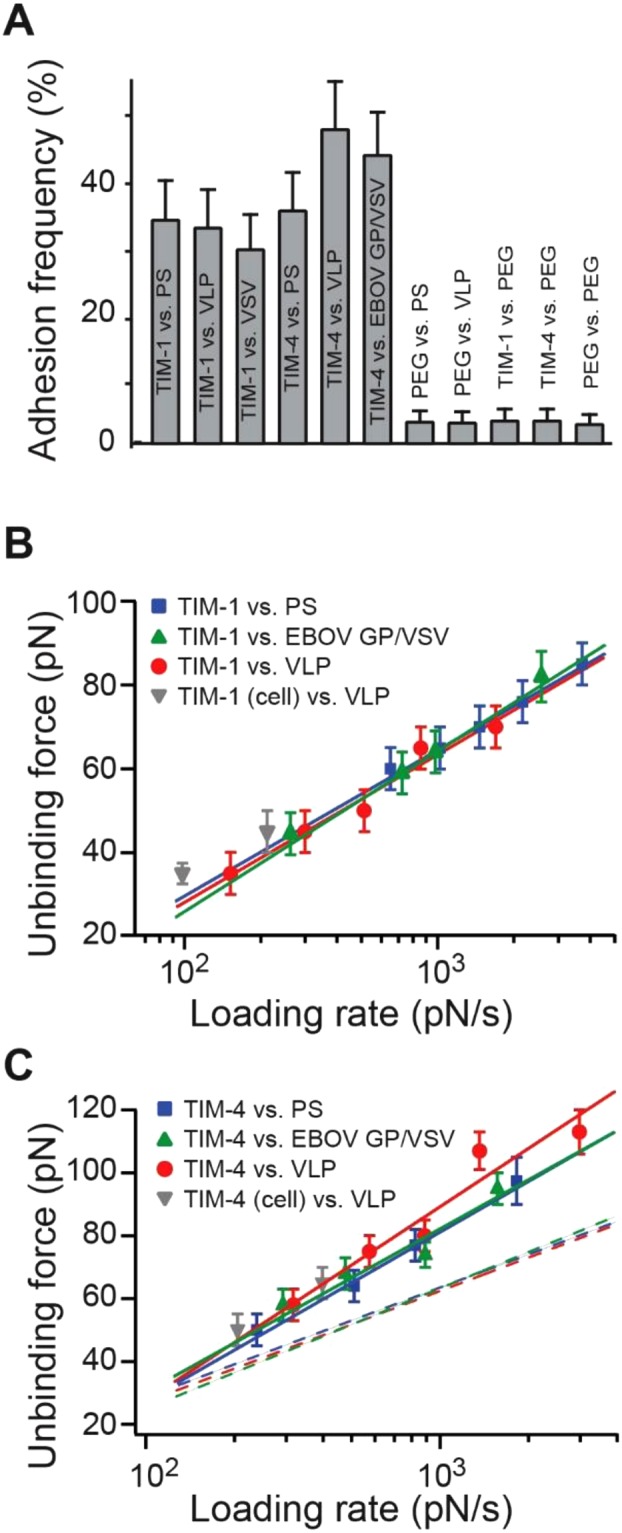
AFM measurement of TIM-ligand interactions. (**A**) The adhesion frequency of the AFM measurements for different interacting pairs. Contact force, contact time and retraction speed for all the interacting AFM tip and surfaces were set at 200 pN, 0.43 s and 1.5 µm/s, respectively. Error bars are Poisson errors (i.e., the square root of the adhesion number). (**B**) The dynamic force spectrum (i.e., the plot of the most probable unbinding force, F_u_, as a function of the loading rate, r_F_) of the TIM-1–PS, TIM-1–VLP, TIM-1 (cell bound)–VLP and TIM-1–EBOV GP/VSV interactions. Unbinding forces at different loading rates were plotted as histograms (Fig. S1). Loading rates were determined directly from the force-extension data by multiplying the system spring constant (Fig. 1B) of the unbinding pulling trace and the retraction speed of the cantilever. The peak of each histogram (i.e., the most probable unbinding force) was plotted against the loading rate; uncertainty in the peak forces is shown as half of the bin width. Solid lines are linear fits to equation (2) for TIM-1–ligand interactions. EBOV GP/VSV is an abbreviation for the EBOV glycoprotein/vesicular stomatitis virus pseudovirions. (**C**) The dynamic force spectra of the TIM-4–PS, TIM-4–VLP, TIM-4 (cell bound)–VLP, and TIM-4–EBOV GP/VSV interactions. Solid lines are linear fits to equation [2] for TIM-4–ligand interactions. Dashed line is the linear fits for TIM-1–ligand interactions taken from (**B**).

Interaction specificity was shown by the adhesion frequency measurement under the same measurement conditions. Fig. 2A shows a significant decrease in adhesion when either the TIM or its binding partner (PS, VLP or EBOV GP/VSV) was absent, confirming that the vast majority of the recorded unbinding force stemmed from specific TIM-ligand interactions.

The biophysical properties of TIM-ligand interactions were studied by the means of a dynamic force spectrum (DFS) (Fig. 2B). The DFS is the plot of most probable unbinding force as a function of the loading rate. The loading rate is obtained by multiplying the system’s spring constant (Fig. 1B) and the pulling speed of each force curve. The unbinding forces of each TIM-ligand interactions were first grouped into 4 to 5 groups by their loading rates. The distribution of forces within the same group was analyzed by histograms (Supplementary Figs S1 and S2). The most probable unbinding forces were then determined from the modes of each histograms. Fig. 2B shows that the unbinding force of the TIM-1–PS, TIM-1–VLP, and TIM-1–EBOV GP/VSV bonds increased linearly with the logarithm of the loading rate, ranging similarly from 40 to 80 pN over a loading rate of 200 to 4,000 pN/s, respectively.

In order to confirm our findings on a cell surface, HEK293T cells were transfected with TIM-1. Under two similar loading rates, the interaction of cell-bound TIM-1 with VLP was mechanically comparable to that when purified soluble TIM-1 was covalently coupled to glass cover slips (Fig. 2B).

A more detailed analysis of the biophysical properties of TIM-1–ligand interactions was conducted by fitting the acquired DFS data to the Bell-Evans model. The model describes the influence of an external force on the rate of bond dissociation^45^. According to this model, a pulling force, f, distorts the intermolecular potential of a ligand-receptor complex, leading to a lowering of the activation energy and an increase in the dissociation rate k(f) as follows:

1 $$k(f)=\frac{1}{t(f)}={k}^{0}\exp (\frac{f\gamma }{{k}_{b}T})$$

where *k*^*0*^ is the dissociation rate constant in the absence of a pulling force, γ is the position of the transition state, T is the absolute temperature, and k_B_ is the Boltzmann constant. For a constant loading rate, r_f_, the model can be described as:

2 $${f}^{\ast }=\frac{{k}_{b}\,T}{\gamma }\,\mathrm{ln}(\frac{\gamma }{{k}^{0}{k}_{b}T})+\frac{{k}_{b}T}{\gamma }\,\mathrm{ln}({r}_{f})$$

hence, as predicted by the model, the most probable unbinding force f* is a linear function of the logarithm of the loading rate. Experimentally, f* was determined from the mode of the unbinding force histograms (Supplementary Figs S1 and S2). Fitting the DFS of TIM-1–PS interaction to the Bell-Evans model (Eq. 2) yielded a dissociation rate in the absence of force (k^0^) of 0.76 s^−1^, and an activation barrier width (γ) (i.e, the distance to the transition state) of 0.28 nm. The fitted curves are overlaid on the DFS, and the best-fit parameters, *k*_*o*_, and γ are tabulated in Table 1. Within fitting uncertainties, the fitted Bell-Evans model parameters for the TIM-1–VLP, and the TIM-1–EBOV GP/VSV interactions are similar to those of the TIM-1–PS interaction.

**Table 1:** Bell-Evans model parameters of the TIM-ligand interaction. Uncertainties are the standard error of the fits. Asterisks indicate significant differences (p < 0.05) between TIM-1 and TIM-4 group values.

*TIM-ligand pairs*	k^0^ (s^−1^)	*τ*^*0*^ (s)	*γ* (Å)
TIM-1 vs. PS	0.76 ± 0.14	1.32 ± 0.25	2.8 ± 0.1*
TIM-1 vs. VLP	1.01 ± 0.29	0.99 ± 0.28	2.7 ± 0.3*
TIM-1 vs. EBOV GP/VSV	1.16 ± 0.18	0.86 ± 0.14	2.5 ± 0.2*
TIM-4 vs. PS	1.21 ± 0.36	0.77 ± 0.10	1.8 ± 0.2*
TIM-4 vs. VLP	1.26 ± 0.50	0.79 ± 0.31	1.6 ± 0.3*
TIM-4 vs. EBOV GP/VSV	0.90 ± 0.45	1.11 ± 0.55	1.9 ± 0.3*

Using a similar approach, we have studied the interactions between TIM-4 and ligands (PS and VLP) (Fig. 2C). Notably, unbinding forces for TIM-4–ligand interactions were higher than those of the TIM-1–ligand interactions (Supplementary Fig. S2), indicating that TIM-4–ligand bonds have greater mechanical strength than TIM-1–ligand bonds. Fitting the DFS to equation (2) indicates that TIM-4–ligand interactions have insignificantly different dissociation rates (all approximately 1 s^−1^), but significantly shorter barriers (around 0.2 nm) compared to those of TIM-1–ligand interactions (Table 1). The shorter barrier width suggests that the TIM-4–ligand bond is more resistant to mechanical pulling. Similarly, Dobrowsky *et al*., reported that a shorter barrier width yielded stronger binding for human HIV gp120–receptor interactions^46^.

In addition to using the Bell-Evans model, we also fitted the unbinding data to the Dudko-Hummer-Szabo model^47^. The distributions of unbinding forces at different loading rates (Supplementary Figs S1 and S2) were first fitted to a statistical model developed by Dudko *et al*.^47^ to obtain the force dependent lifetimes (Supplementary Fig. S3). The average lifetimes for six different TIM-ligand interactions were then fitted to the Dudko-Hummer-Szabo model (Supplementary Fig. S3). The fitted results were summarized in Table 2. Consistently, the Dudko-Hummer-Szabo model fit yielded comparable zero-force lifetimes (τ^0^) among all the tested TIM-ligand interactions, and that TIM-4–ligand bonds had shorter distance to transition state compared to TIM-1–ligand bonds. Notably, compared to the Bell-Evans model, the Dudko-Hummer-Szabo model showed a 2- to 5- fold greater zero-force lifetimes (τ^0^) (Tables 2 and 3).

**Table 2:** Dudko-Hummer-Szabo model parameters of the TIM-ligand interaction. Uncertainties are the standard error of the fits.

	*τ*^*0*^ (s)	*Δx* (Å)
TIM-1 vs. PS	2.8 ± 2.5	4.0 ± 0.9
TIM-1 vs. VLP	3.7 ± 2.1	4.2 ± 0.7
TIM-1 vs. EBOV GP/VSV	3.3 ± 2.4	4.4 ± 1.0
TIM-4 vs. PS	3.8 ± 2.4	3.3 ± 0.6
TIM-4 vs. VLP	3.2 ± 2.8	2.6 ± 0.9
TIM-4 vs. EBOV GP/VSV	3.5 ± 2.5	2.7 ± 1.0

**Table 3:** Continuum model variables and parameters.

Variables and Parameters	Definition	Value	Normalized Variable
*β*	Free energy of each TIM/PS bond	$17\,{k}_{B}T$	
*R*	EBOV radius	$40\,nm$	
$\rho$	Density of TIM bonds	$1000\,\mu {m}^{-1}$	
$\kappa$	Cell membrane bending rigidity	$40\,{k}_{B}T$	
$a$	Half of the EBOV/host cell contact width		$\bar{a}=\frac{a}{R}$
$l$	Half of the characteristic distance over which adhesion occurs		$\bar{l}=\frac{l}{R}$
$b$	$b=l-a$		$\bar{b}=\frac{b}{R}$
δ	EBOV indentation depth		$\bar{\delta }=\frac{\delta }{R}$
$F$	External force		$\bar{F}=\frac{F}{\rho \beta }$
${U}_{total}$	Total energy (per unit length out of plane)		${\bar{U}}_{total}=\frac{{U}_{total}}{\rho \beta R}$
$\alpha$	Normalized bending constant		$\alpha =\frac{\kappa }{2\rho \beta {R}^{2}}$

**Figure 3:**
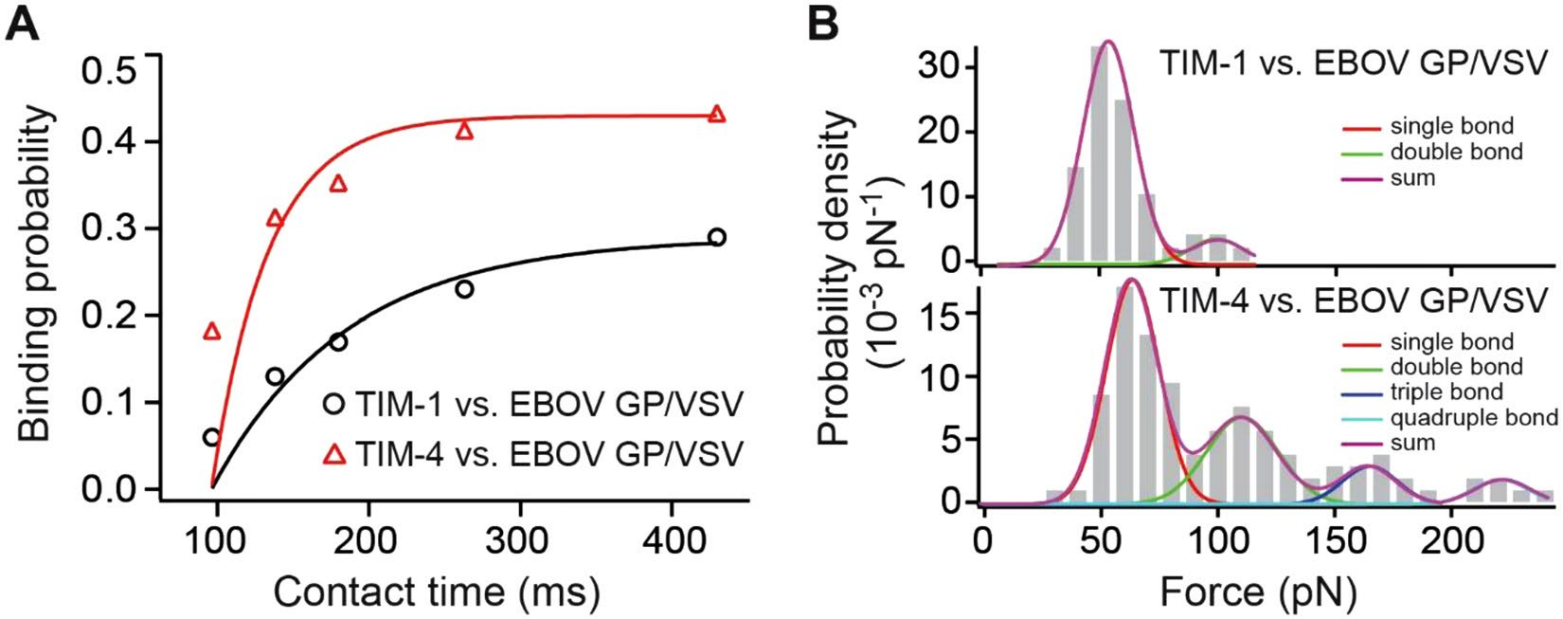
On-rate measurements. (**A**) The binding probabilities (P) of TIM-1–EBOV GP/VSV and TIM-4–EBOV GP/VSV interactions are plotted as a function of the contact time (t). The solid line is the result of least-squares fits of equation P = A(1 − (1−exp(−(t − t_0_)/τ)), where A and t_0_ are the maximal observed binding probability and the shortest contact time tested, respectively. The binding time constant is τ. Equation k_on_ = 1/(τC_eff_) was used to estimation the on-rates. (**B**) Unbinding force distributions of TIM-1–EBOV GP/VSV (upper panel) and TIM-4–EBOV GP/VSV (lower panel) interactions at 430 ms contact time. Multiple-peak Gaussians were fitted to the curves. Each Gaussian peak and the sum of fitted Gaussians are overlaid on the distribution curve.

The kinetics for a receptor-ligand interaction (i.e., bonding and de-bonding) is characterized by the interaction’s on- and off-rates. We have estimated on-rates (k_on_) of TIM-1−EBOV GP/VSV and TIM-4−EBOV GP/VSV interactions, using a method established by the Hinterdorfer group^23,48^. By assuming the interactions follow a pseudo first-order kinetics, the on-rates can be estimated using k_on_ = (τ C_eff_)^−1^, where τ refers to the interaction time, and C_eff_ denotes the effective concentration of the binding partner (in this case TIM-1 or TIM-4 on the surface) within an effective volume, V_eff_. The V_eff_ can be approximated by the volume of a sphere, whose radius r_eff_ is the sum of the size of the virus and cross-linker^48^. Therefore, the equation can be rewritten as: k_on_ = N_A_·(4/3)·π·r_eff_3·n_b_^−1^·τ^−1^, where N_A_ is the Avogadro constant, and n_b_ is the number of the binding partner^48^. Fig. 3A shows binding probabilities (P) of TIM-1–EBOV GP/VSV and TIM-4–EBOV GP/VSV interactions as a function of contact time (t). The P vs. t curves were fitted to a monoexponential decay function, yielding the time constant τ of the interaction at 89 ± 18 ms for TIM-1 and 37 ± 17 ms for TIM-4, respectively. n_b_ can be estimated from probability density curves for unbinding forces at the longest contact time^23^. Shown in Fig. 3B, at a contact time of 430 ms, both TIM-1 and TIM-4 unbinding forces showed multimodal distributions. Multiple Gaussian peak analyses showed two peaks for TIM-1–EBOV GP/VSV interactions: at 58 and 101 pN, and four peaks for TIM-4–EBOV GP/VSV interactions: at 69, 116, 170, and 221 pN. Therefore, the available binding partners for the EBOV GP/VSV to engage, estimated by the maximum bond numbers, were two and four for TIM-1 and TIM-4, respectively. Finally, using the 107.8 nm reported size of VSV^49^, and the 3 nm cross-linker length^23^, the k_on_ is estimated as 1.9 × 10^7^ M^−1^s^−1^ for TIM-1, and as 2.2 × 10^7^ M^−1^s^−1^ for TIM-4. Using the k^0^_off_ values estimated from the Bell-Evans model (Table 1), the equilibrium dissociation constant K_d_, was then estimated to be approximately 50 nM for both TIM-1–EBOV GP/VSV and TIM-4–EBOV GP/VSV interactions. If using the kinetics values estimated from the Dudko-Hummer-Szabo model (Table 2), the K_d_ would be approximately 15 nM. These two K_d_ values are comparable with the 24 nM K_d_ reported by Rankl *et al*.^23^ for human rhinovirus − host cell interactions.

**Figure 4:**
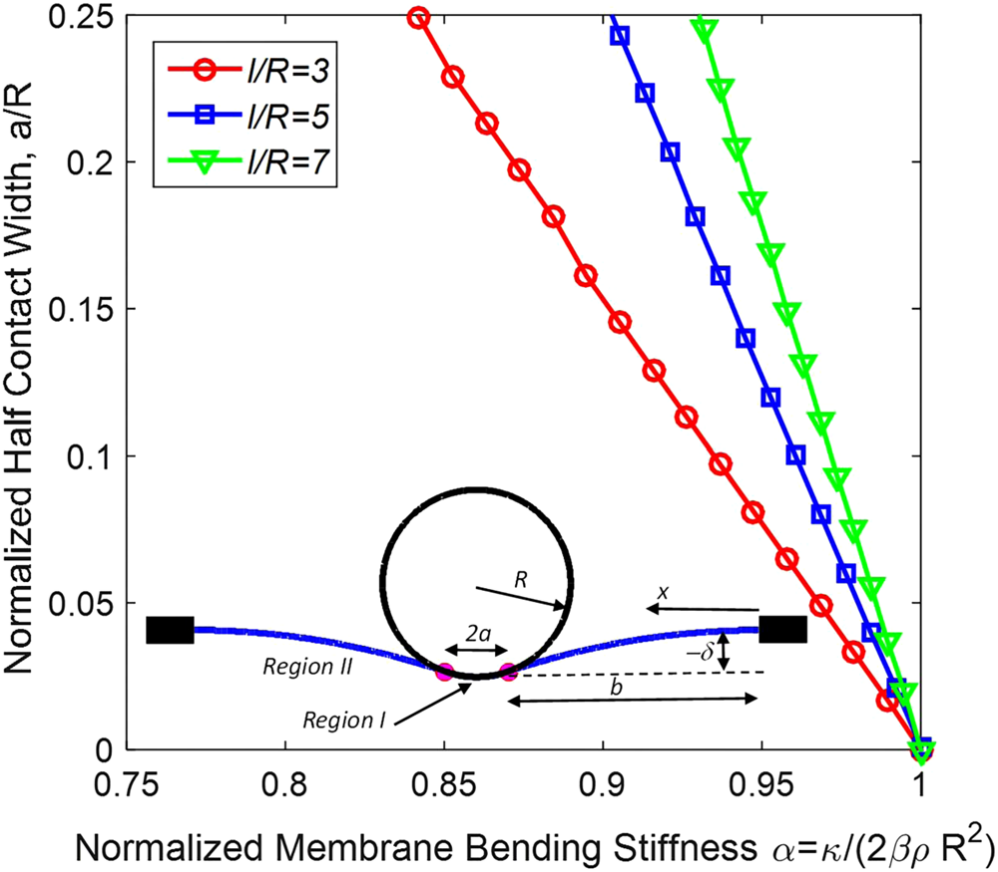
Mechanical model of EBOV-host cell attachment driven by adhesion and resisted by membrane bending. Our principal result is that if a dimensionless parameter representing the ratio of bending and adhesion energies has value greater than one, then there is no adhesion. If it assumes values less than one, contact width grows rapidly, leading to strong adhesion.

How do the biophysical parameters identified from single-molecule studies relate to the mechanics of virus adhesion? To address this question, we have developed a simple model for EBOV-host cell interaction driven by adhesive interactions between the virus and cell-membrane receptors. The model parameters are summarized in Table 3. The inset in Fig. 4 shows the model schematically. The virus is assumed to be cylindrical and relatively stiff so that it maintains its circular cross-section. Because its length can be large compared to the radius, we model the interaction in the two-dimensions of the virus cross-section. The viral particle is about 80 nm in diameter, much smaller than the size of the cell it infects. Therefore, we assume that the cell membrane (Fig. 4, blue line) is originally flat but deforms under contact with the virus. The contact width is *2a* and the cell membrane is supported some distance *l* = *a* + *b* away from the center of the virus attachment point; this represents a characteristic distance over which macropinocytosis occurs.

Physically, attachment of the virus to the cell membrane is driven by adhesion between the two and we assume it is resisted by energy required to bend the cell membrane. (We assume that the membrane tension is sufficiently small such that bending dominates over tension. In Supporting Information, we provide a quantitative criterion for this condition, as well as results for the limit where tension dominates over bending.) To express this mathematically, we write the total energy of the system as a sum of contributions from the elastic bending of the membrane and adhesion between the membrane and viral particle (by symmetry, we model only the right half of the region shown in Fig. 4):

3 $${U}_{total}={U}_{elastic}+{U}_{adhesive}={U}_{I}+{U}_{II}+{U}_{adhesive}$$

where *U*_*elastic*_ is the contribution due to membrane bending, and ${U}_{adhesive}$ is the contribution due to adhesion between TIM and the viral surface^33,50^. The elastic bending energy is a sum of contributions from two regions, region I where the membrane is in contact with the virus and region II where it is free of lateral loads. The deflection *w(x)* is prescribed in region *I* by the circular shape of the virus cross-section and, according to small-deflection plate theory^51^ *w(x)* is governed in region *II* by the differential equation

4 $$\kappa \frac{{d}^{4}{w}_{II}}{d{x}^{4}}=0$$

The elastic energy (per unit length out of plane) in terms of the displacements is given by

5 $${U}_{elastic}={\int }_{b}^{l}\frac{\kappa }{2}{({w^{\prime\prime} }_{I})}^{2}dx+{\int }_{0}^{b}\frac{\kappa }{2}{({w^{\prime\prime} }_{II})}^{2}dx$$

where $\kappa$ is the bending rigidity of the cell membrane (in Joules; typically 10–100 k_B_T^52–54^). The adhesion energy is given by

6 $${U}_{adhesive}=-\,\rho (l-b)\beta $$

where $\rho$ is the number of bonds per unit area, and $\beta$ is the free energy of binding of each bond. We proceed as follows. (See SI for details.) For specified values of parameters *b*,*l*,*δ*,*R*,*β*, and $\kappa$, we solve equation [4] subject to the conditions that deflection matches the circular profile of the virus in region *I*; *w* and *w'* are both zero at *x* = 0; and both are continuous at *x* = *b*. We then impose a condition of configurational energy balance, that is, energy is minimized with respect to location of the contact edge:

7 $$\frac{d{U}_{total}}{db}=0$$

The minimum energy condition provides the equilibrium value of *b* (and hence of *a*) eliminating *b* as a variable. Finally, we vary *δ* until the value of the net normal force, which is proportional to *w''* evaluated at *x=b*, equals zero. This situation corresponds to adhesion of the virus to the cell membrane in the absence of a net external force, and thus eliminates *δ* as a variable.

The following normalization simplifies the analysis and reveals the important dimensionless parameters that govern the adhesion behavior. We normalize all distances and sizes by the radius of the virus:

8 $$\bar{\delta }=\frac{\delta }{R};\,\bar{b}=\frac{b}{R};\,\bar{l}=\frac{l}{R}$$

and force and energy as

9 $$\bar{F}=\frac{F}{\rho \beta };\,{\bar{U}}_{total}=\frac{U}{\rho \beta R}$$

With normalization we find that the solution depends solely on two dimensionless parameters:

10 $$\alpha =\frac{\kappa }{2\rho \beta {R}^{2}}\,{\rm{and}}\,\bar{l}$$

(This dimensionless parameter has previously been identified by Deserno^33,50^). In particular, the equilibrium contact width,$\,\bar{a}=\bar{a}(\alpha ,\bar{l})$. The first parameter, $\alpha$, represents the ratio of bending energy ($\kappa /{2}$) for one radian and adhesion energy $\rho \beta {R}^{2}$.

Figure 4 shows results for normalized half contact width versus normalized membrane bending stiffness for three different values of $\bar{l}$. The most important and definitive conclusion of our analysis is that a condition for any adhesion between the virus particle and the cell is:

11 $$\alpha =\frac{\kappa }{2\rho \beta {R}^{2}} < 1$$

This condition is *necessary* regardless of the value of $\bar{l}$. If this condition is not met, the equilibrium contact width is zero; there is no adhesion. The second important conclusion is that for relevant values of $\bar{l}$, the normalized contact width rises rapidly with decrease of membrane stiffness below the value needed to have any adhesion. We show results only over a range of contact width for which the linear Euler-Bernoulli beam model used here should be accurate. However, it does show the importance of the parameter $\alpha$: if it exceeds unity there is no adhesion; if it is only somewhat smaller than unity, the contact width increases rapidly.

In order to connect this result with the force-spectroscopy measurements, we estimate *β* in terms of the dissociation constant (for the usual reference concentration of 1 Mol/L)

12 $$\beta =-\,{k}_{B}T\,ln({K}_{d})$$

which yields a value of about 17 ${k}_{B}T$ when using the 50 nM K_d_ estimated from single-molecule experiments for both TIM-1/PS and TIM-4/PS interactions. The EBOV virus has a radius of about 40 nm. Therefore the range of *l/R* used in Fig. 4 matches the range of expected dimensions. Picking reasonable values for $\rho =1000\,per\,sq.\mu m\,$^55^, $\kappa$ = 40 *k*_*B*_*T*^56^; *R = 40 nm*, we find *α* = 0.69, i.e., having a value sufficiently small to result in strong adhesion.

In SI we present an analysis of the limiting case in which tension dominates over bending. If *T* is the tension in the membrane, we show that if the parameter, $\gamma =\frac{T}{\rho \beta } < 1$, we predict strong adhesion of the viral particle.

## Discussion

Here, using single-molecule force spectroscopy, we have quantified the mechanical strength between the following TIM-ligand bonds: TIM-1–PS, TIM-4–PS, TIM-1–EBOV GP/VSV, TIM-1–VLP, TIM-4–VLP, and TIM-4–EBOV GP/VSV. Our DFS data revealed that under the loading rates ranging from 200 to 4,000 pN/s, unbinding forces ranged, respectively, from 40 to 80 pN for TIM-1–ligand bonds, and from 50 to 100 pN for TIM-4–ligand bonds. The loading rate range was chosen based on the estimated physiological loading rates (i.e., 125 to 2500 pN/s.) of cellular tethered bonds in the vasculature^57^. Similar level of unbinding forces within the loading rates have been reported for several adhesion receptor-ligand systems such as integrin-ligand, selectin-ligand, and antibody-antigen interactions, suggesting that the mechanical strength of TIM-ligand bond is comparable to these adhesion molecule-ligand interactions that are known to withstand mechanical loads. In line with this observation, TIM-1 has been shown to interact with P-selectin and mediate T-lymphocyte tethering and rolling on vascular endothelium^58^.

Both TIM-1 and TIM-4 are known to mediate EBOV entry via binding of PS^15,16^. However, a discrepancy exists as to whether TIM-1 or TIM-4 also binds with EBOV surface GP^59^. For both TIM-1 and TIM-4, the DFS among TIM-PS, TIM-VLP, and TIM-EBOV GP/VSV are almost indistinguishable (Fig. 2). This observation is consistent with previous force spectroscopy studies of single virus−host cell interactions. For instance, Rankl *et al*.^23^ reported similar reaction kinetics (i.e., k_on_, k_off_, and γ) for the interactions between human rhinovirus and two forms of low-density lipoprotein receptor: cell membrane-bound and soluble recombinant. Consistently, Chang, *et al*.^60^ and Dobrowsky *et al*.^46^ conducted single-molecule analyses of the interactions between type-1 human immunodeficiency virus gp120 and T-cell CD4 receptor. The authors observed similar reaction kinetics in both transmembrane and soluble forms of CD4. In addition, since the both the VLPs and EBOV GP/VSVs used here express GP, and the DFS provides a biomechanical signature of different TIM-ligand interaction, our data suggests that TIM-1 and TIM-4 interact with the PS on the VLP or EBOV GP/VSV, not the GP.

**Figure 5:**
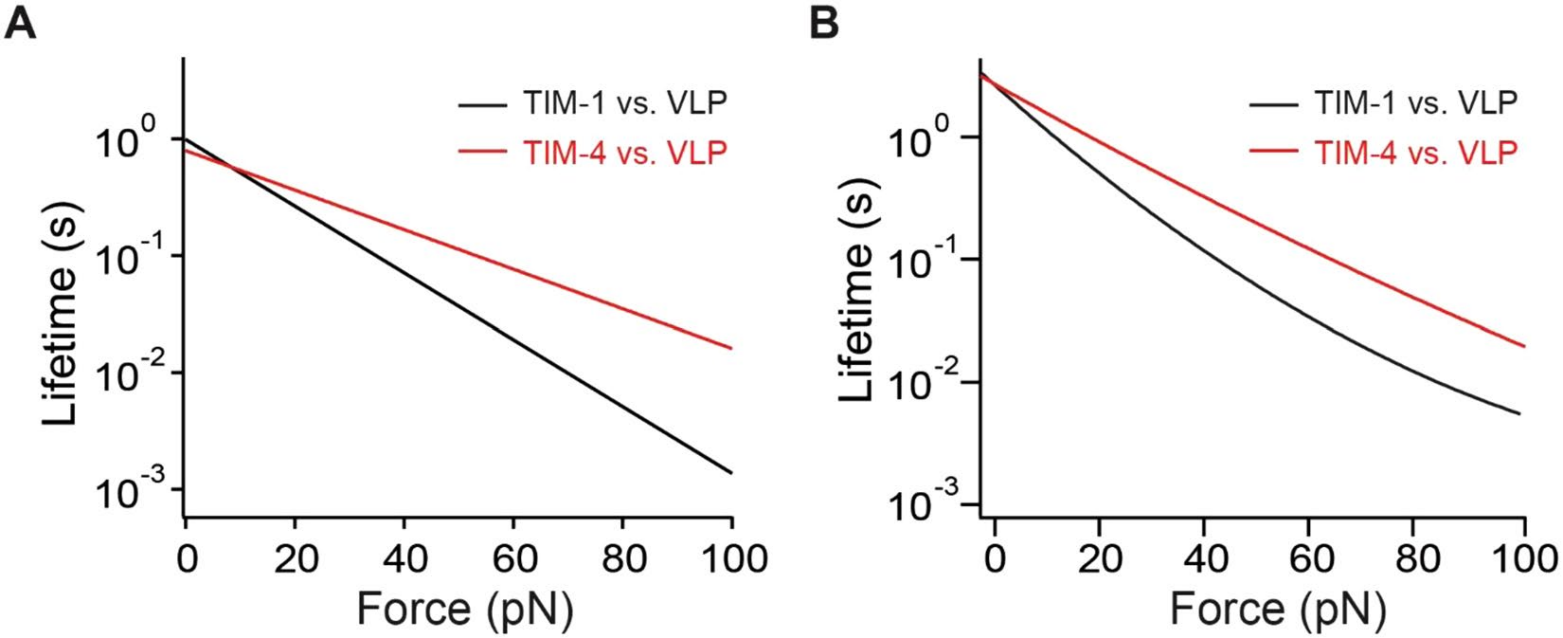
(**A**) The force-dependent lifetimes of TIM-1–VLP and TIM-4–VLP bonds given by Bell-Evans model fit (Equation 1). (**B**) The force-dependent lifetimes of TIM-1–VLP and TIM-4–VLP bonds given by Dudko-Hummer-Szabo model fit (taken from Supplementary Fig. S3).

Studies suggest that both murine and human TIM-1 serve as the most important plasma membrane receptors for EBOV in epithelial cells^12^. EBOV can also infect dendritic cells and macrophages, causing disseminated intravascular coagulation^61,62^. Due to its expression on some macrophages, TIM-4 is likely a membrane receptor for EBOV^16,20^ on these cells. Our study provides an estimation of the effects of pulling force on bond lifetimes (Fig. 5). A comparison of the lifetimes of the TIM-1–VLP and TIM-4–VLP bonds, given by both Bell-Evans and Dudko-Hummer-Szabo models, consistently revealed that the lifetimes for the two bonds are comparable under no force (Fig. 5). However, when force is applied, TIM-4–VLP bond exhibits greater mechanical resistance and longer lifetime than the TIM-1–VLP bond. At higher force (60–100 pN), the differences of lifetime between the bonds are close to, or exceed, one order of magnitude (Fig. 5). Therefore, the TIM-4 is more suitable than TIM-1 to mediate attachment of EBOV under mechanical disturbance. Since TIM-4 expresses on leukocytes (such as macrophages) that may be exposed to larger forces in the blood, the higher force resistance of TIM-4–ligand interaction could help EBOV to remain adherent to host cells in the vasculature.

Both TIM-1 and TIM-4 interact with PS through their amino terminal (N-terminal) immunoglobulin variable (IgV)-like domain^16,63^. The PS-binding pocket is located between two loops of the IgV domain^64,65^, the upper FG and the lower CC’ loop, which form a conserved pocket known as a metal ion-dependent ligand-binding site (MILIBS)^66^. The stronger mechanical strength in TIM-4–ligand interaction may be explained by a cooperative binding among TIM-4’s PS-binding residues when they interact with PS. A recent study suggests that TIM-4 has more IgV residues interacting with EBOV than those of TIM-1^16^. Tietjen *et al*. has reported that these additional PS-binding residues located outside the MILIBS promote cooperative binding of TIM-4 to PS^67^, which could explain the enhancement of mechanical strength in TIM-4–PS complex. To understand the contributions of different structural components of IgV on the mechanical strength of the binding, further force spectroscopy studies using various IgV mutants^11,16^ of TIM-1 and TIM-4 are needed.

We have developed a simple model for the mechanics of attachment of viral particles to a cell membrane. The purpose of the model is to show how single molecule measurements can be combined with other physical properties of the system, such as density of ligand-receptor pairs and membrane stiffness, to predict whether and to what extent a viral particle will adhere to the cell membrane. We model attachment as being driven by TIM–PS adhesion and resisted by membrane bending. (In some cells, membrane tension will be the dominant player resisting deformation.) The parameters that govern the process of adhesion in this model: adhesion free energy *β*, viral radius *R*, bending stiffness *k*, length *l*, tension, *T*, TIM surface density *β*, combine into three dimensionless groups. These are *α*, a ratio of bending and adhesion energies; *γ*, a ratio of tension and adhesion energy, and a normalized distance. When resistance to deformation is dominated by bending, if *α* exceeds unity in value, our continuum model predicts that no adhesion is possible. That is, membrane bending stiffness (and/or tension) can act as an agent blocking adhesion. For values of *α* less than one, the size of the contact increases rapidly, leading to strong adhesion of the apoptotic or viral particle. If tension dominates over bending as the agent resisting deformation, the process of adhesion is dominated by the value of a single parameter representing the ratio of works of tension and adhesion. In order to connect the mechanical model to the AFM experiments, we show that viral particle adhesion to the membrane occurs for TIM-ligand adhesion free energy calculated using values of ${k}_{on}$ and ${k}_{off}$ determined experimentally. Because experiments have established that ${K}_{D}$ is very small (<<mM), the probability of association of even a single bond is nearly unity. Thus, our model shows that the interplay between adhesion and bending energies sets up equilibrium states that either have association probability of essentially ‘1’ (α < 1) or ‘0’ (α > 1).

Although we are not aware of direct experimental evidence for the predicted blocking effect of membrane stiffness (or tension), there is significant indirect evidence of the kind of adhesive process we have modeled^34^. See Fery *et al*.^68^ for direct visual evidence of adhesion between a bead and a vesicle; Malsam *et al*.^69^ and LeBihan *et al*.^70^ for evidence of adhesion between vesicles. Several micrographs in Hernandez *et al*.^71^ indicate equilibrium shapes of the sort we have calculated, evidently based on balance between an adhesive driving force and resistance either by bending or tension.

Our theoretical model uses a continuum description of the main physical agents: membrane bending stiffness, distributed adhesion energy, and tension. By its simplicity, it has allowed us to highlight the importance of two dimensionless groups of parameters and their potential ability to block adhesion. By the same token, our model suffers from limitations, addressing which will require more complex models. For example, in the continuum model, we identify vanishing contact width with lack of adhesion whereas it is conceivable that a single receptor-ligand pair of sufficient strength^57^ might tether the virus to the cell membrane for long enough to initiate processes leading to engulfment. (A line of contacting pairs is deemed unlikely given the distribution of TIM location on the cell membrane). That would represent a breakdown of the continuum assumptions we have made. The model also does not explicitly account for thermal fluctuations or the discrete nature of receptor-ligand interactions. The former means, for example, that the coupling between tension and bending fluctuations is not captured; the latter can have a significant effect on effective work of adhesion^72,73^ if individual ligand-receptor strength is strong but their surface density is small.

In conclusion, the study shows the biomechanical parameters important for Ebola attachment to host cells. The study has demonstrated experimentally that TIM-EBOV interactions are mechanically comparable to adhesion molecule (e.g., selectin)−ligand interactions. Through a simple mechanical model, we further demonstrate how molecular binding parameters determine whether they are sufficient for viral adhesion. The study may provide new information to aid in the development of new antiviral therapeutics for the prevention and treatment of EBOV disease.

## Materials and Methods

### Cell culture

Immortalized HEK 293 T cells purchased from American Type Culture Collection (ATCC) were cultured in DMEM medium (ATCC), and supplemented with 4 mM L-glutamine, 4500 mg/L glucose, 1 mM sodium pyruvate, 1500 mg/L sodium bicarbonate, 1% penicillin streptomycin, and 10% fetal bovine serum. The cells were grown in T-25 flasks (Corning) at 37 °C in a 5% CO_2_ atmosphere and plated for experiments in 35 mm Cyto-One culture dishes (USA Scientific).

### VLP generation

EBOV GP-pseudotyped VLPs were generated by co-transfecting HEK 293 T cells with a plasmid expressing EBOV VP40 fused to green fluorescent protein (GFP)^74^, and a plasmid expressing EBOV GP, at a 1:1 ratio^15^. Supernatants were collected 48 and 72 h after transfection. VLPs were concentrated by centrifuging supernatants overnight at 5,380 × g at 4 °C. Pellets were resuspended in 1 × PBS and purified through a sucrose cushion. Pellets were resuspended in 1 × PBS, filtered through a 0.45-μm syringe filter, aliquoted, and stored at −80 °C.

### EBOV GP/VSV pseudovirion generation

As previously described^15^, vesicular stomatitis virus (strain Indiana) virions with genomes in which the G glycoprotein gene is replaced with enhanced green fluorescent protein (EGFP), HEK 293 T cells were transfected with plasmids expressing EBOV GP and transduced 24 h later with EBOV GP/VSV ΔG-EGFP pseudovirions. After 4 h of virus uptake, the plates were washed, and medium was repleted. Pseudotyped virions were collected in supernatant 48 and 72 h following transduction, pooled, and filtered through a 0.45-μm filter. The pseudovirions were concentrated by centrifuging supernatants at 5,380 × *g* overnight at 4 °C and resuspending pellet in fresh medium to achieve higher-titer stocks.

### TIM-1 and TIM-4 Transfection

The HEK 293 T cells were split the day before transfection in 35 mm Cyto-One culture dishes (US Scientific) at 50% confluence. 200 µl of Opti-MEM (1×) reduced serum medium (Life Technologies) was added to a 0.5 ml tube. 1 µg of TIM-1 or TIM-4 expression plasmid^15,16^ was transfected into the cells using MegaTran 1.0 transfection reagent (Origene Technologies) with a ratio of 1:3 (DNA:MegaTran) according to manufacturer’s instructions.

### Cantilever preparation/ coverslip preparation

To functionalize AFM cantilevers (MLTC, Bruker Nano) with PS, the cantilever was first silanized with 3-(trimethoxysilyl)propyl methacrylate to obtain surface thiol groups. Phosphatidylserine (PS)-PEG-Maleimide (NANOCS) was coupled to the cantilever^23^. VLPs^15,16^ of EBOV, or EBOV GP/VSV were immobilized onto a (3-aminopropyl)-triethoxysilane sinalized AFM cantilever (MLTC, Bruker Nano) using a heterobifunctional polyethylene glycol (PEG) crosslinker, Acetal-PEG-NHS (Institute of Biophysics, Johannes Kepler University), according to the protocol developed by Dr. Hermann J. Gruber, Johannes Kepler University (http://www.jku.at/biophysics/content)^23^. Soluble recombinant TIM-1 or TIM-4 (R&D Systems) was attached to the silanized glass coverslips using the same crosslinking approach. Functionalized cantilevers and glass surfaces were stored in PBS (3 × 5 min) and used for AFM experiment within 8 hours.

### Single-molecule force measurements

All single-molecule force measurements were conducted using a custom-designed AFM apparatus. AFM measurements were collected at cantilever retraction speeds ranging from 0.19 to 3.7 µm/s to achieve the desired loading rate (200–4,000 pN/s) or 1.53 to 3.7 µm/s when the proteins were being separated from HEK 293 T cells. This was necessary to compensate for softness of the cell surface, which would act to lower the loading rate. All measurements were conducted at 25 °C in Tris-buffered saline (TBS), supplemented with 5 mM CaCl_2_. The contact time and indentation force between the cantilever and the sample were minimized to obtain measurements of the unitary unbinding force.

### Statistical Analysis

For each pulling speed, over 500 force curves were recorded, which yielded 40 to 200 unbinding forces. Curve fitting was performed using IGOR Pro or Origin software by minimizing the chi-square statistic for the optimal fit. Unless otherwise stated, the data is reported as the mean and the standard error of the estimate. Statistical analyses between groups were performed using an unpaired t-test or ANOVA, with a p-value less than 0.05 considered to be statistically significant.

## Electronic supplementary material

Supplementary figures and modeling information
