# Supplementary material for: Biomechanical characterization of TIM protein–mediated Ebola virus–host cell adhesion
Source: Sci Rep. 2019 Jan 22;9:267. doi: 10.1038/s41598-018-36449-2 (PMC6342996; doi:10.1038/s41598-018-36449-2)
Supplement: Supplementary file 1 — Supplementary figures and modeling information [file 41598_2018_36449_MOESM1_ESM.pdf]

Supporting Information

Biomechanical characterization of TIM protein–mediated Ebola virus–host cell adhesion

by

Matthew A. Dragovich[1,#], Nicole Fortoul[2], Anand Jagota[2, 3], Wei Zhang[1], Krista Schutt[3,##], Yan Xu[1], Michelle Sanabria[3], Dennis M. Moyer Jr. [3], Sven Moller-Tank[4], Wendy Maury[4], and X. Frank Zhang[1, 3,*]

[1]Department of Mechanical Engineering & Mechanics, [2]Department of Chemical and Biomolecular Engineering and [3]Bioengineering Program, Lehigh University, 19 Memorial Drive West, Bethlehem, PA 18015, USA.

[4]Department of Microbiology, University of Iowa, Iowa City, IA 52242, USA

Part I. Supplemental figures

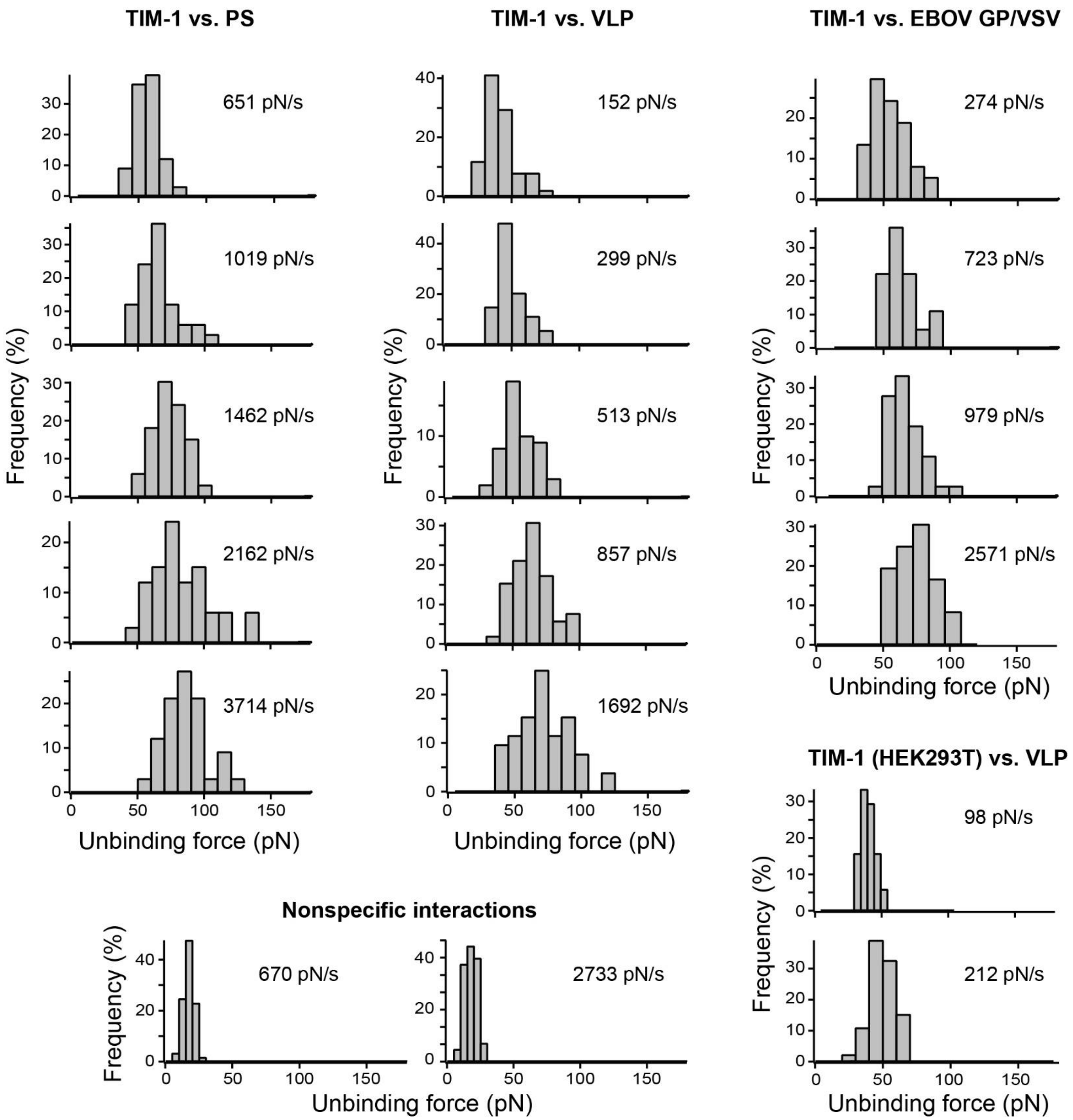

Figure S1. Unbinding force histograms of TIM-1–ligand interactions at different loading rates. Loading rates were determined directly from each force-displacement curve by multiplying the system spring constant of the unbinding trace and the retraction speed of the cantilever. Nonspecific interactions (lower left panel, occurred at an adhesion frequency of 4.7%) were recorded between a PEG-modified AFM tip and a TIM-1 modified surface. Specific TIM-1–ligand unbinding forces are defined as forces >25 pN, determined by the averaged nonspecific force plus one SD. Specific forces occurred at adhesion frequencies of ~30%. The modes of each specific force histogram were recorded as most probable unbinding forces for Bell-Evans model analysis (Fig. 2B).

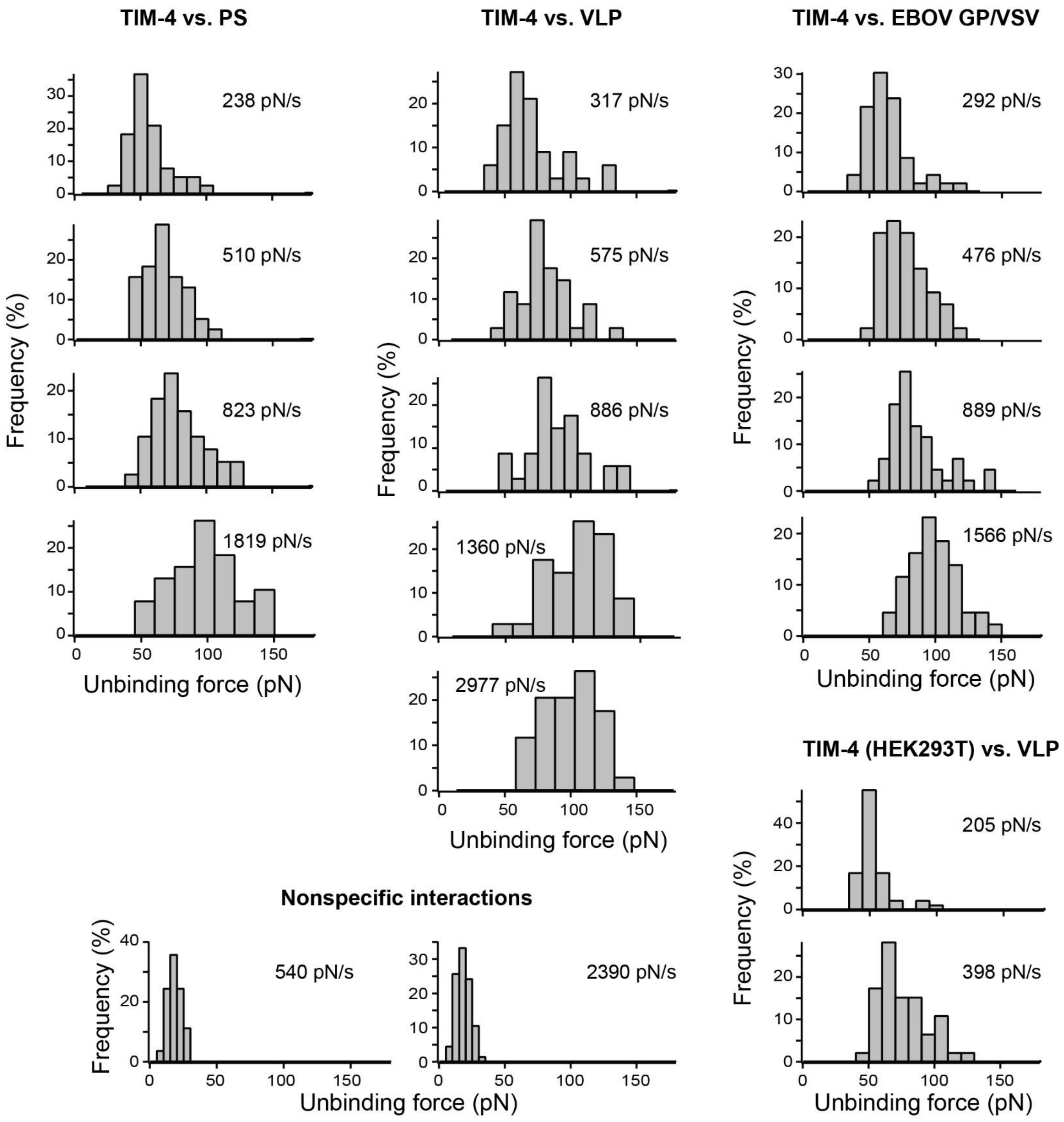

Figure S2. Unbinding force histograms of TIM-1–ligand interactions at different loading rates. Loading rates were determined directly from each force-displacement curve by multiplying the system spring constant of the unbinding trace and the retraction speed of the cantilever. Nonspecific interactions (lower left panel, occurred at an adhesion frequency of 4.9%) were recorded between a PEG-modified AFM tip and a TIM-4 modified surface. Specific TIM-4–ligand unbinding forces are defined as forces >25 pN, determined by the averaged nonspecific force plus one SD. Specific forces occurred at adhesion frequencies of ~30%. The modes of each specific force histogram were recorded as most probable unbinding forces for Bell-Evans model analysis (Fig. 2C).

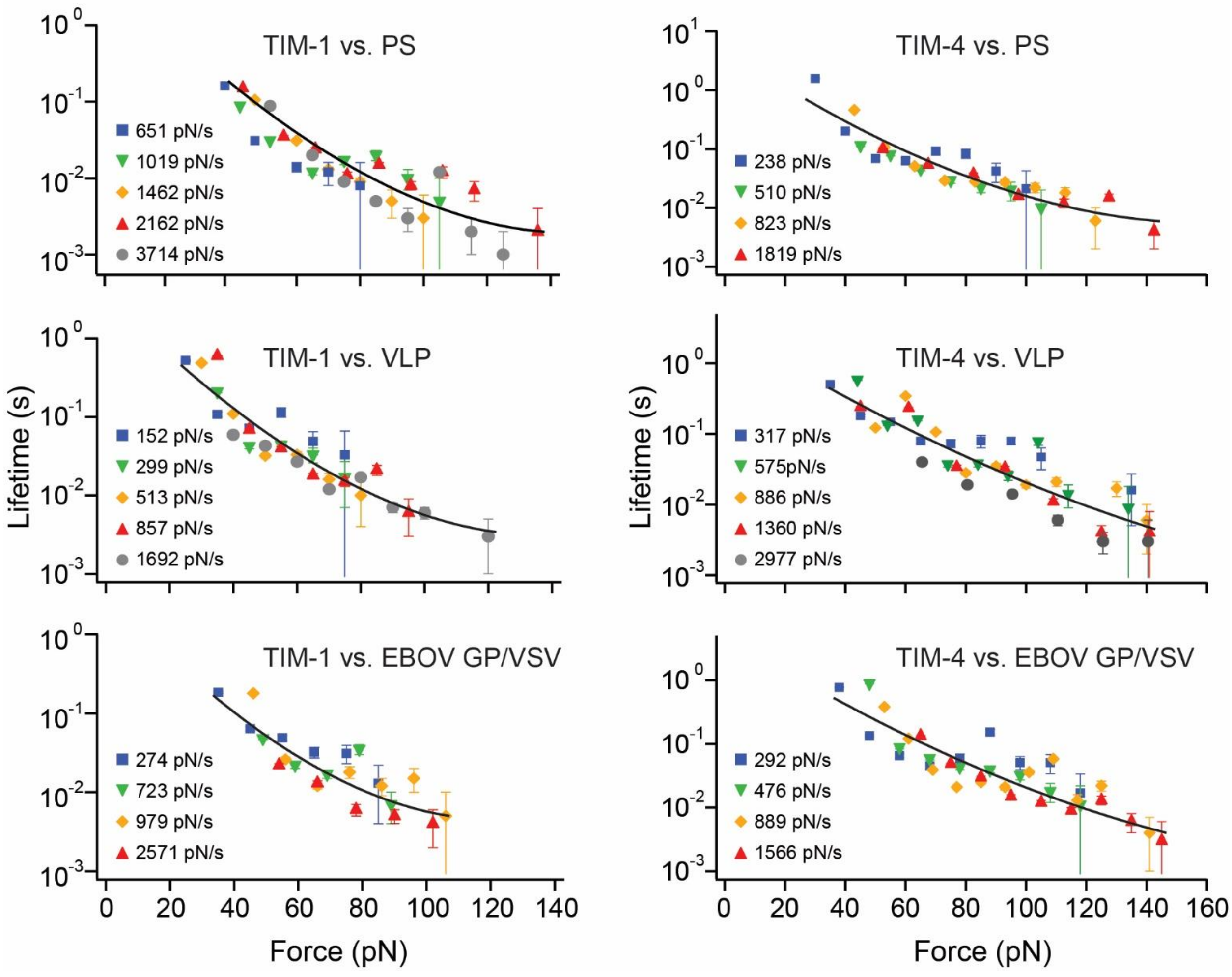

Figure S3. Lifetimes (τ) for TIM-ligand interactions as a function of the applied force F, obtained by transforming the histograms of unbinding forces (Figs. S1 and S2) using a statistical method developed by Dudko, et al. [1]. The histogram has N bins of width $\Delta F = (F_{max} - F_{min})/N$. Let the number of counts in the ith bin be $c_i, 1 \le i \le N$, then the total counts is $C = \sum_{i=1}^{N} c_i$, resulting in the probability $P(F_i) = c_i/C$, and the probability density $p(F_i) = c_i/(C \cdot \Delta F)$. Thus, the force-dependent lifetime is: $\tau(F_i) = \frac{\left(p(F_i)/2 + \sum_{k=i+1}^{N} p(F_i)\right)\cdot \Delta F}{\dot{F}(F_i)\cdot p(F_i)}$, where $F_i = F_{min} + (i - 1/2)\Delta F$. The lifetimes were then fitted with the Dudko-Hummer-Szabo model equation:

$$\tau(F) = \tau_0 \left(1 - \frac{\nu F \Delta x^{\ddagger}}{\Delta G}\right)^{1-1/\nu} \cdot \exp\left\{-\beta \Delta G \left[1 - (1 - \frac{\nu F \Delta x^{\ddagger}}{\Delta G})^{1/\nu}\right]\right\}$$

Here $\tau^0 = 1/k^0$, and ν is a scaling factor and was set as 1/2, which specifies the free-energy surface has the cusp shape. Δx is the distance to the transition state. The fitted results are summarized in Table 2. The error bars are the Poisson errors of the lifetime. Some error bars are within the symbol.

Part II. Supplementary modeling information

S2.1 Mechanics of Virus Attachment by Adhesion

Our model is based on interaction between the viral particle and cell membrane driving adhesion, which is at the energetic cost of deforming the membrane. Generally, the agents resisting deformation are either bending of the membrane, its tension, or both. In the main text of the manuscript we present results based on the assumption that bending dominates over tension. To quantify this condition, consider the equation governing deflection *w* of the membrane when both bending and tension are present:

$$\kappa \frac{d^4 w}{dx^4} - T \frac{d^2 w}{dx^2} = 0 \qquad \text{(A1)}$$

where *T* is the tension (N/m) and κ is the bending rigidity,

In normalized form, this is

$$\frac{\kappa}{TR^2} \frac{d^4 \bar{w}}{d\bar{x}^4} - \frac{d^2 \bar{w}}{d\bar{x}^2} = 0 \qquad \text{(A2)}$$

Thus, we can neglect tension if $\frac{\kappa}{TR^2} \gg 1$; conversely we can neglect bending if $\frac{\kappa}{TR^2} \ll 1$. Using values for $\kappa = 40\ k_B T$ and *R*=40 nm, we find $T<100\ pN/\mu m$ is needed to be able to neglect tension. Later in this SI, we present a derivation that applies when tension dominates over bending. First, we present details of the derivation in the limit when bending dominates.

S2.2 Mechanics of Virus Attachment by Adhesion When Bending Dominates over Tension

In that case, the governing equation for membrane deformation is

$$\frac{d^4 \bar{w}}{d\bar{x}^4} = 0 \qquad \text{(A3)}$$

We analyze the geometry shown in Figure A, and adopt the following additional assumptions:

1. The membrane acts as if it is on a fluid substrate for x=0 to x=l
2. The membrane is tethered at the ends a distance *b* from where the viral particle touches the membrane
3. The virus is rigid and nondeformable
4. By symmetry about the center axis of the virus, we model only the right half of the geometry.

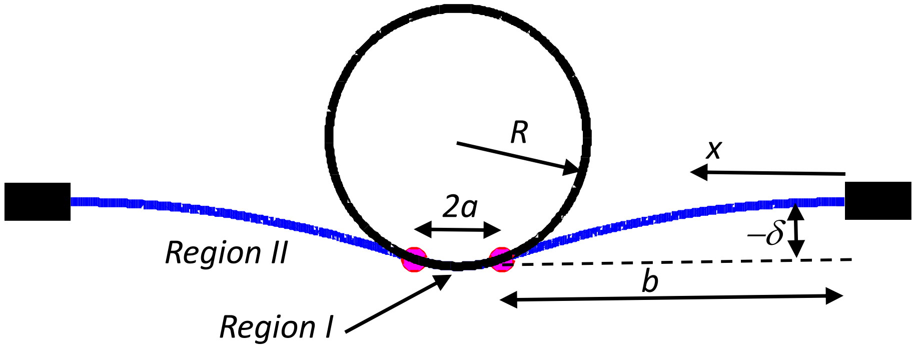

Figure S4. Geometry of cylindrical viral particle adhering to a cell membrane.

Region I (where the virus adheres to the membrane)

Membrane deflection in this adhesive zone between x=b and x=l=a+b is prescribed by the shape of the viral particle:

$$y = R - \sqrt{R^2 - (l - x)^2} \qquad \text{(A4)}$$

Using Taylor Series Expansion, this approximates to $\frac{x^2}{2R}$. The deflection in region I is therefore

$$w_I(x) = \delta + \frac{(l-x)^2}{2R} \qquad \text{(A5)}$$

$$w_I'(x) = -\frac{(l-x)}{R} \qquad \text{(A6)}$$

At the boundary between regions I and II,

$$w_I(b) = \delta + \frac{(l-b)^2}{2R} \qquad \text{(A7a)}$$

$$w_I'(b) = -\frac{(l-b)}{R} \qquad \text{(A7b)}$$

Region II (Membrane not in contact with virus)

Integrating the governing equation (A3), we get

$$w_{II}(x) = c_1 + c_2 x + c_3 x^2 + c_4 x^3 \qquad \text{(A8)}$$

$$w_{II}'(x) = c_2 + 2c_3 x + 3c_4 x^2 \qquad \text{(A9)}$$

This must satisfy the boundary conditions:

$$w_{II}(b) = w_I(b) = \delta + \frac{(l-b)^2}{2R} \quad \text{(A10a)}$$

$$w_{II}(0) = 0 \quad \text{(A10b)}$$

$$w'_{II}(b) = w'_I(b) = -\frac{(l-b)}{R} \quad \text{(A10c)}$$

$$w'_{II}(0) = 0 \quad \text{(A10d)}$$

(There is no cumulative force in region I and II, but there is a point force at the boundary between the two regions.) This gives us the result that $c_1 = c_2 = 0$, and

$$c_3 = \frac{b^2+6\delta R-4bl+3l^2}{2Rb^2} \quad \text{(A11)}$$

$$c_4 = \frac{-2\delta R+(b-l)l}{Rb^3} \quad \text{(A12)}$$

Now that we know the deflection everywhere, we can create the expression for U_{total} (under displacement control)

$$U_{total} = U_{elastic} + U_{adhesive} = U_I + U_{II} + U_{adhesive} \quad \text{(A13)}$$

$$U_{total} = \int_b^l \frac{\kappa}{2}(w''_I)^2\,dx + \int_0^b \frac{\kappa}{2}(w''_{II})^2\,dx - \rho(l-b)\beta \quad \text{(A14)}$$

$$U_{total} = \frac{\kappa}{2}\left(\frac{1}{R}\right)^2(l-b) + \frac{\kappa}{2}(4bc_3^2 + 12c_4^2b^3 + 12c_3c_4b^2) - \rho\beta(l-b) \quad \text{(A15)}$$

In terms of normalized variables:

$$\bar{\delta} = \frac{\delta}{R}$$

$$\bar{b} = \frac{b}{R}$$

$$\bar{c_3} = Rc_3$$

$$\bar{c_4} = R^2c_4$$

$$\bar{l} = \frac{l}{R}$$

$$U_{total} = \frac{\kappa R}{2}\left(\frac{1}{R}\right)^2(\bar{l}-\bar{b}) + \frac{\kappa}{2R}(4\bar{b}\bar{c}_3^2 + 12\bar{c}_4^2\bar{b}^3 + 12\bar{c}_3\bar{c}_4\bar{b}^2) - \rho\beta R(\bar{l}-\bar{b}) \quad \text{(A16)}$$

Further defining normalized variables:

$$\bar{F} = \frac{F}{\rho\beta}$$

$$\alpha = \frac{\kappa}{2\rho\beta R^2}$$

$$\bar{U}_{total} = \frac{U}{\rho\beta R}$$

We find

$$\bar{U}_{total} = \alpha\left(\bar{l} - \bar{b}\right) + \alpha(4\bar{b}\bar{c}_3^2 + 12\bar{c}_4^2\bar{b}^3 + 12\bar{c}_3\bar{c}_4\bar{b}^2) - \left(\bar{l} - \bar{b}\right) \quad \text{(A17)}$$

First we (numerically) find the equilibrium point, i.e., the value of $\bar{b}$ for which $\frac{d\bar{U}_{total}}{d\bar{b}} = 0$. Next, we vary $\bar{\delta}$ until we find the condition where force on the membrane is zero. This is done by evaluating shear force

$$V = -\frac{dM}{dx} = -\frac{\kappa\, d^3w}{dx^3} = -6c_4$$

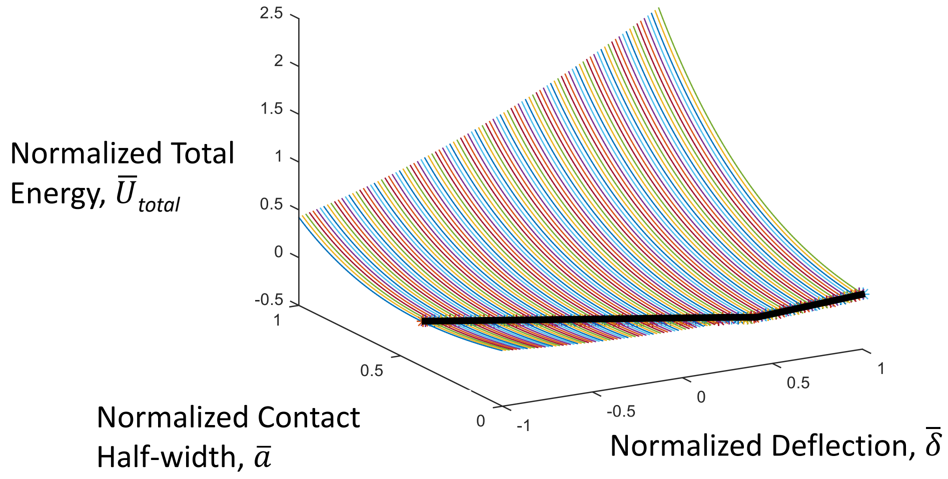

Figure S5. Example of normalized total energy as a function of normalized deflection and contact-half-width for $\alpha = 0.85$ and $\bar{l} = 5$. For each deflection, we find the value of normalized contact half-width that minimizes total energy. Then, along the locus of these points (black line in the figure), we search for the condition that shear force is zero.

S2.3 Mechanics of Virus Attachment by Adhesion: Limiting Case of High Tension

In the main text, it is assumed that bending of the membrane dominates and the tension in the membrane is negligible. As a limiting case, we also study the effect of high tension and negligible bending energy of the membrane. As before, we are interested in the case where there is no external force applied on the system. In that case, one can assume by force balance that the non-contacting region of the membrane is horizontal (x=0 to x=b) because for a membrane the force on its cross-section is tangential to the membrane surface.

The adhesive energy between the virus and the membrane, $U_{adhesive}$, can be represented by

$$U_{adhesive} = -R\theta\rho\beta \quad \text{(A18)}$$

where θ is the angle of virus internalization ($\theta = 0$ representing no adhesion, and $\theta = \pi$ corresponding to total engulfment). The work done by the tension in the membrane, $W_{tension}$, equals the product of tension and total length:

$$W_{tension} = T(R\theta + l - Rsin\theta) \quad \text{(A19)}$$

Adding the energy contributions from the tension in the membrane to the adhesive energy from the TIM-1 interaction we find the total energy to be

$$U_{total} = TR(\theta + \bar{l} - sin\theta) - \frac{TR\theta\rho\beta}{T} \quad \text{(A20)}$$

which can be nondimensionalized to

$$\bar{U}_{total} = \frac{U_{total}}{TR} = \theta + \bar{l} - sin\theta - \frac{\theta}{\gamma} \quad \text{(A21)}$$

using the dimensionless variable γ that represents the ratio between tension an adhesion

$$\gamma = \frac{T}{\rho\beta}$$

Setting the derivative of the energy to zero identifies the equilibrium condition:

$$\frac{d\bar{U}_{total}}{d\theta} = 1 - cos\theta - \frac{1}{\gamma} = 0$$

which yields the relation

$$cos\theta = 1 - \frac{1}{\gamma} \quad \text{(A22)}$$

The relationship between γ and θ helps one to determine the following conditions:

1. If $\gamma = 1 \rightarrow \theta = \pi/2$, Tension is equal to adhesion and half of the virus is adhered to the membrane
2. If $\gamma \gg 1 \rightarrow \theta = 0$, Tension dominates, the virus is just touching the membrane, and no adhesion occurs
3. If $= \frac{1}{2} \rightarrow \theta = \pi,$ Adhesion dominates.
4. If $< \frac{1}{2}$, Adhesion dominates.

References:

1 Dudko, O. K., Hummer, G. & Szabo, A. Theory, analysis, and interpretation of single-molecule force spectroscopy experiments. *Proc Natl Acad Sci U S A* **105**, 15755-15760, doi:10.1073/pnas.0806085105 (2008).
